# Hemorrhage and complications associated with percutaneous ultrasound guided liver biopsy in dogs

**DOI:** 10.1111/jvim.15942

**Published:** 2020-10-30

**Authors:** Jonjo Reece, Michelle Pavlick, Dominique G. Penninck, Cynthia R. L. Webster

**Affiliations:** ^1^ Cummings School of Veterinary Medicine at Tufts University Grafton Massachusetts USA

**Keywords:** coagulation, hepatic, packed cell volume, ultrasound

## Abstract

**Background:**

Liver biopsy is often necessary to obtain a diagnosis in dogs with hepatobiliary disease. Hemorrhage after biopsy is a concern.

**Objective:**

To describe the extent of hemorrhage and incidence of complications after percutaneous ultrasound guided liver biopsy (PUGLB) in dogs and to examine risk factors for hemorrhage or complications.

**Animals:**

One hundred two client owned dogs with suspected hepatobiliary disease that underwent PUGLB.

**Methods:**

Medical records were retrospectively reviewed. Using human guidelines, major hemorrhage was defined as an absolute decrease in the PCV (ΔPCV) ≥ 6%. Complications were defined separately as clinically relevant physiologic compromise that necessitated intervention or death. The relationship between ΔPCV and the occurrence of complications and the initial PCV, coagulation variables, serum activity of liver‐derived enzymes, serum bilirubin concentration, number of biopsies, biopsy needle gauge, radiologist experience, histological diagnosis, and ultrasound variables were compared.

**Results:**

Before PUGLB, most aberrations in coagulation variables were mild. After biopsy a decrease in PCV occurred in 87/102 (85.3%) dogs. The mean ΔPCV was −7.2% ± 4.5%. Major hemorrhage occurred in 43/102 (42.2%) dogs and complications in 2/102 (1.9%). ΔPCV was significantly positively correlated with PCV before biopsy (*r* = .47, *P* = .004). There was no correlation between ΔPCV or complications with any of the variables examined.

**Conclusion and Clinical Importance:**

Percutaneous ultrasound guided liver biopsy in the population of dogs in the current study, with normal or mild abnormalities in coagulation, results in a high incidence of clinically silent, major hemorrhage (42.5%), but few complications (1.9%).

**Abbreviations**
ALT: alanine aminotransferase
aPTT: activated partial thromboplastin time
AST: aspartate aminotransferase
GGT: gamma glutamyltranspeptidase
PT: prothrombin time
PUGLB: percutaneous ultrasound guided liver biopsy
TS: total solids
ULN: upper limit of normal

## 1 INTRODUCTION

Liver histopathology is necessary to diagnose and treat hepatobiliary disorders in dogs.^1^, ^2^ A sample can be obtained by percutaneous ultrasound guided liver biopsy (PUGLB) using needle biopsy techniques or by punch, cup or wedge biopsy at laparoscopy or laparotomy.^1^, ^2^ The most frequent complication of PUGLB in humans is hemorrhage.^3^, ^4^ Hemorrhage requiring intervention occurs in 0.4% of adults on average (range, 0%‐5.3%).^5^, ^6^, ^7^, ^8^ In children, the incidence of major hemorrhagic complications is greater and occurs in 2% of children on average (range, 0%‐4.6%).^9^, ^10^, ^11^

The incidence of hemorrhage after PUGLB in the dog has not been well characterized. In 1 study of 310 percutaneous ultrasound guided biopsies of thoracic and abdominal organs in dogs, the complication rate for minor (drop in PCV of >10% that did not require any intervention or caused no measurable compromise in the dog) or major (required intervention) hemorrhage in dogs was 18.5% and 4.2%, respectively.^12^ Since the number of liver biopsies was not articulated in the article, it is not possible to determine the incidence of complications with liver biopsy alone. However, 3/13 (23%) dogs with major complications had a liver biopsy.^12^ In a study of 195 dogs that underwent ultrasound guided biopsies or fine needle aspirates of abdominal organs, a low incidence of both major complications (3/246, 1.2%), including bile peritonitis and hemorrhage, and, minor complications (13/246, 5.6%) associated with minor localized hemorrhage were noted.^13^ Another study reported no complications after PUGLB in dogs, but did not monitor the PCV after biopsy.^14^

Since the liver is the site of synthesis and clearance of most pro and anticoagulants, as well as, the regulators of fibrinolysis, assessment of hemostasis is recommended prior to a liver biopsy.^1^, ^2^, ^3^, ^4^, ^15^ Conventional plasma based coagulation testing which includes prothrombin time (PT), activated partial thromboplastin time (aPTT) and platelet count are advocated for before biopsy to assess risk of hemorrhage in humans.^3^, ^4^, ^16^, ^17^, ^18^, ^19^ Severe thrombocytopenia (<50 000/μL), moderate to severe prolongations in prothrombin time (PT) (>1.5 to 2.0‐fold) and low fibrinogen levels (<60 mg/dL) predict risk of hemorrhage in human patients with cirrhosis.,^3^, ^20^, ^21^, ^22^, ^23^, ^24^ However, other studies suggest that these conventional coagulation tests do a poor job in predicting risk of hemorrhage.^17^, ^18^, ^19^ In a single veterinary study in dogs, a PT > the upper limit of the reference range or a platelet count <80 000/μL were risk factors for complications due to hemorrhage after ultrasound guided biopsy of abdominal and thoracic organs.^12^

Risk factors, other than coagulation variables, for major hemorrhage after PUGLB in humans include age (younger and older are at a greater risk), body weight in children, female sex, more than 3 passes with the needle, cirrhosis, neoplasia, and presence of anemia before biopsy.^4^, ^7^, ^11^, ^16^, ^17^, ^18^, ^19^ A single veterinary study has examined risk factors for percutaneous biopsy in dogs. This study which looked at ultrasound guided biopsies of all abdominal and thoracic organs suggested that a greater number of passes and the presence of non‐neoplastic lesions were risk factors.^12^

The aim of this current study was to describe the extent of hemorrhage and incidence of complications after PUGLB in dogs and secondarily to identify possible risk factors for hemorrhage.

## 2 MATERIALS AND METHODS

Medical records at Foster Hospital for Small Animals, Cummings School of Veterinary Medicine at Tufts University, between 2002 and 2015, were searched for dogs that underwent PUGLB. Dogs were included if they had a PUGLB performed, a coagulation profile (PT, aPTT, and platelet count) that was obtained within 24 hours before PUGLB, a PCV that was obtained within 24 hours prior to the procedure, at least 1 PCV that was obtained within 36 hours after the biopsy and a medical record available for review. Dogs were excluded if they had any treatments that might have altered the PCV, such as, transfusion or fluid resuscitation, or were given a drug that might alter hemostasis (nonsteroidal anti‐inflammatory medications, corticosteroids, heparin, clopidogrel, free fatty acids, or hydroxylethyl starch). Charts were reviewed for the following: signalment, PCV before biopsy, total solids (TS), PT, aPTT, platelet count, serum activity of liver‐derived enzymes [alanine aminotransferase (ALT), alkaline phosphatase (ALP)], serum albumin, serum bilirubin concentration, PCV, and TS after biopsy, expertise of the radiologist performing the PUGLB, (radiology resident or board‐certified radiologist), biopsy needle size, number of biopsies obtained, adequacy of the biopsy sample, histopathologic diagnosis, anesthesia protocol, and additional procedures done. The PCV and TS were obtained at variable intervals after biopsy and the lowest value within 36 hours after biopsy and the time at which the lowest value occurred were recorded. The records were reviewed to determine if additional procedures were performed on the same day of the liver biopsy.

Since there are no established guidelines in veterinary medicine to classify the extent of hemorrhage after procedures, we used definitions from the human literature.^22^, ^23^, ^24^, ^25^ Although the criteria we chose were defined for human patients on antihemostatic medications, they have been extensively applied to assess hemorrhage in human patients with liver disease and were recently applied to cats.^6^, ^8^, ^11^, ^23^, ^26^, ^27^ Under these guidelines major hemorrhage is defined as an absolute decrease in the hemoglobin of 2 g/dL. A fall in hemoglobin of 2 g/dL corresponds with a 6% decrease in PCV, therefore, an absolute decrease in the PCV of 6% was used as a cutoff to define major hemorrhage. The absolute (PCV_pre_ − PCV_post_) change in PCV and the relative (PCV_pre_ − PC_post_/PCV_pre_) decrease in PCV (ΔPCV and RΔPCV, respectively) were calculated.

Complications were defined independently from hemorrhage. A complication was defined as a physiologic compromise necessitating an intervention, or death. Medical records were reviewed to determine if an intervention (such as resuscitative fluids or a blood transfusion) was needed due to hemodynamic instability (increased heart rate, hypotension, depression).

A board‐certified radiologist or a radiology resident performed the PUGLB. An 8‐5 MHz microconvex transducer was used. The biopsies were performed using an automated, double spring‐loaded biopsy instrument (BARD MAGNUM Biopsy Gun, Covington, Georgia). Depending on liver size and the radiologist's assessment of a safe window for biopsy, either an 18‐gauge or 16‐gauge Tru‐cut biopsy needle was used. Biopsies were usually procured from the lateral aspect of the left side of the liver after color Doppler evaluation, to ensure a safe location without visible large vessels.

## 3 STATISTICAL ANALYSIS

All data were analyzed for normality using tests for kurtosis and skewness. Data were expressed as median and range (nonparametric) or mean and SD (parametric). PCV before biopsy, clinicopathologic variables (serum activity of liver‐derived enzymes, bilirubin concentration, PT, aPTT, platelet count), the number of biopsies, biopsy needle gauge, radiologist experience, histological diagnosis, and ultrasound variables were compared between dogs with and without major hemorrhage and between dogs with and without complications using the Fisher's exact test. Correlations were assessed using Pearson's correlation coefficient for continuous variables (after log transformation if necessary) and chi‐square test for discrete variables. A *P* value <.05 was considered significant. Statistics were completed using an online program (https://www.socscistatistics.com).

## 4 RESULTS

The search from 2002 to 2015 identified 102 dogs that underwent PUGLB and fulfilled the inclusion criteria. The mean age at the time of biopsy was 9.3 years (range, 1‐14 years). There were 85 pure breed dogs (83.3%) and 17 mixed breed dogs (16.6%). There were 11 Labradors, 6 Golden Retrievers, 5 German Shepherds, 5 Jack Russell Terriers and 54 other breeds. There were 59 male dogs (57.8%) and 43 female dogs (42.2%). Within our study population, 92.3% of the dogs were neutered. Weight was available in 75/102 dogs. The mean weight was 17.6 kg ± 12 kg.

All dogs had coagulation testing and a biochemical profile before biopsy. Selected clinicopathologic variables are summarized in Table 1. Before biopsy, 26/102 (25.5%) dogs were anemic (PCV <36%) with a median PCV of 32% (14%‐35%) and 16/102 (15.6%) were thrombocytopenic (<150 000/μL) with a median platelet count of 117 000/μL (45 000‐148 000/μL). The PT and aPTT were prolonged in 11/102 (10.6%) and 5/102 (4.9%) dogs respectively. The median prolongations of PT and aPTT were 1.2 (range, 1.03‐1.65) and 1.04‐fold (range, 1.01‐1.15) above the upper limit of normal respectively. Eighty‐seven of 102 (85.3%) dogs had a decrease in PCV after biopsy. The mean absolute ΔPCV was −7.2% ± 4.5%. The mean RΔPCV was 12.8% ± 13.2%. ΔPCV was significantly positively correlated with PCV before biopsy (*r* = .47; *P* = .004). Twenty‐nine dogs with normal PCV prior to the biopsy became anemic after biopsy with a median PCV of 34% (25%‐35%) after biopsy. The average ΔPCV in these dogs was −9.7% ± 4.7%.

**TABLE 1:** Selected clinicopathologic variables in dogs before undergoing PUGLB (n = 102)

Clinicopathologic variable	Median (range)	# Increased	# Decreased	Reference range
PCV (%)	43 (14‐59)	2/102	29/102	39‐55
Total solids (g/dL)	6.8 (4‐10)	19/102	5/102	5.5‐7.8
PT (s)	7.8 (5.8‐15.4)	12/102	2/102	6.9‐9.3
aPTT (s)	11.8 (8.1‐18.2)	5/102	3/102	8.9‐16.3
Platelet (× 10^9^/L)	291 (45‐743)	13/102	17/102	173‐486
Bilirubin (mg/dL)	0.2 (0‐33)	33/99	0/99	0.1‐0.3
ALT (U/L)	308 (25‐5111)	84/101	0/101	14‐86
ALP (U/L)	646 (10‐8484)	84/100	0/100	12‐127
Albumin (g/dL)	3.4 (1.6‐4.5)	8/101	22/101	1.6‐4.5

Abbreviations: ALP, alkaline phosphatase; ALT, alanine aminotransferase; aPTT, activated partial thromboplastin time; PT, prothrombin time; PUGLB, percutaneous ultrasound guided liver biopsy; ULN, upper limit of normal.

Major hemorrhage occurred in 43/102 (42.2%) dogs. The mean ΔPCV in dogs with and without major hemorrhage was −10.8% ± 3.6% and − 3.7% ± 1.8%, respectively. The mean RΔPCV in dogs with and without major hemorrhage was 25% ± 14.4% and 10.8% ± 3.6%, respectively. The median time to the lowest PCV after biopsy in dogs with and without major hemorrhage was 4.8 hours (range, 1‐18 hours) and 4 hours (1‐29 hour), respectively. Seventeen out of 102 dogs had either no change (5/17) or an increase in PCV (12/17) after biopsy. The median increase in PCV of the 12 dogs after biopsy was 2% (2‐10%).

Conventional coagulation variables were compared in dogs with and without major hemorrhage. There was no significant difference (*P* = .76) in the incidence of PT prolongation in dogs without major hemorrhage (6/59, 10%) and those with major hemorrhage (5/43, 12%). Similarly, there was no significant difference (*P* = .30) in the incidence of prolongation in aPTT in dogs without major hemorrhage (1/59, 2%) and those with major hemorrhage (4/43, 9%). There was also no significant difference (*P* = .18) in the incidence of thrombocytopenia in dogs without major hemorrhage (7/59, 12%) and those with major hemorrhage (10/43, 23%).

Selected clinicopathologic variables in dogs with and without major hemorrhage are presented in Table 2. There was no significant difference between dogs with and without major hemorrhage with respect to age, sex, body weight, total solids, serum albumin, and bilirubin concentration or serum activity of liver‐derived enzymes. PCV before biopsy was significantly (*P* < .001) higher in dogs with major hemorrhage. The mean PCV before biopsy was in the normal range for both groups. Seven of 43 (16%) dogs with major hemorrhage were anemic before biopsy compared to 22/59 (37%) without major hemorrhage. This difference was not significant.

**TABLE 2:** Comparison of clinicopathologic variables in dogs with major hemorrhage (n = 42) and without major hemorrhage (n = 43) after PUGLB (n = 102)

Clinicopathologic variable	Without major hemorrhage median (range)	With major hemorrhage median (range)	*P* value	Reference range
Age (y)	10.5 (1.2‐15.6)	10.1 (2.3‐14.3)	.78	NA
Sex (M:F)	21:38	20:23	.42	NA
Weight (kg)	13.6 (3.6‐49)	10.2 (3.6‐40.2)	.62	NA
PCV (%)	40 (14‐59)	46 (22‐57)	<.001	39‐55
PT × ULN	1.0 (1‐1.27)	1.0 (1‐1.65)	.91	NA
aPTT × ULN	1.00	1.0 (1‐1.15)	.95	NA
Total solids (g/dL)	6.7 (4‐10)	7.0 (4‐9.2)	.35	6.0‐8.4
Platelet (× 10^9^/L)	300 (45‐718)	287 (54‐743)	.12	180‐525
Bilirubin (mg/dL)	0.20 (0.1‐9.2)	0.25 (0.1‐32.4)	.06	0.1‐0.3
ALT (IU)	316 (28‐2149)	385 (41‐3490)	.34	14‐86
ALP (IU)	647 (10‐5764)	833 (34‐3931)	.47	12‐127
Albumin (g/dL)	3.4 (1.5‐4.5)	3.3 (1.9‐4.5)	.55	2.8‐4.0
Number of liver biopsies	3 (2‐5)	3 (1‐6)	.64	NA

Abbreviations: ALP, alkaline phosphatase; ALT, alanine aminotransferase; aPTT, activated partial thromboplastin time; PT, prothrombin time; PUGLB, percutaneous ultrasound guided liver biopsy; ULN, upper limit of normal.

Twenty‐nine of 102 (28.4%) dogs received vitamin K prior to biopsy. A variety of vitamin K dosage regimens were used including 1 mg/kg subcutaneously every 24 hours (7/19, 37%), 1 mg/kg subcutaneously once (3/19, 16%) and 0.5 mg/kg subcutaneously every 12 hours (4/19, 21%). Of the dogs receiving vitamin K, 12/29 (41%) had major hemorrhage and 17/29 (59%) did not have major hemorrhage. There was no significant difference in the incidence of hemorrhage between dogs that did or did not receive vitamin K.

Ninety‐five out of 102 dogs (93.4%) had abnormalities on ultrasound including 34/102 (33.3%) with nodules, 29/102 (28.4%) with a hyperechoic liver, 25/102 (24.5%) with ascites, 16/102 (15.7%) with a focal mass and 8/102 (7.8%) with a hypoechoic liver. Hepatic biopsy was performed by a board‐certified radiologist in 29/102 (28.4%) dogs and by a radiology resident in 73/102 (71.5%) dogs. An 18‐gauge needle was used in 64/102 (62.7%) dogs, a 16‐gauge needle was used in 37/102 (36.3%) dogs and the needle size was not documented in 1 dog. All dogs were sedated for the procedure with 86/102 (84.3%) dogs receiving propofol alone or in combination with an opiate, with or without a benzodiazepine. Two dogs received only an opiate and a benzodiazepine, with 1 of the dogs also receiving ketamine. For 14 dogs the type of sedation was not documented. Dogs had a median of 3 (range, 1‐5) liver biopsy samples. Twenty‐nine of 102 (27.9%) dogs had a concurrent fine needle aspirate or biopsy performed the same day. Fourteen of 102 (13.7%) had fine needle aspirates of the spleen, 3/102 (2.9%) had a kidney biopsy, 2/102 (1.9%) had a splenic biopsy and 2/102 (1.9%) had a splenic aspirate and a splenic biopsy. Other aspirate and biopsy sites included the prostate, abdominal lymph nodes, skin, bone marrow and submandibular lymph node. None of the dogs had a concurrent cholecystocentesis.

There was no significant difference in the number of liver biopsies, biopsy needle gauge, anesthetic protocol, and the number and type of concurrent procedures in dogs, ultrasound findings (nodules, masses, echogenicity, presence of ascites) or experience of ultrasonographer in dogs with and without major hemorrhage.

Two of 102 dogs (1.9%) had a complication. One dog was thrombocytopenic (54 000/μL), anemic (PCV 22%) and had a prolonged PT (1.05 × upper limit of normal) and aPTT (1.15 × upper limit of normal) before liver biopsy. One dog with major hemorrhage required resuscitative fluids due to a decrease in PCV from 51% to 35% and tachycardia after liver biopsy. Coagulation variables were normal in this dog.

Dogs with and without complications had no significant difference between coagulation variables, serum bilirubin concentrations, serum activity of liver‐derived enzymes, ultrasound variables (echogenicity, presence of a mass, presence of nodules, presence of ascites), experience of the radiologist, number and type of concurrent procedures or number of liver biopsies procured.

The biopsy sample was judged to be adequate to describe a histopathologic diagnosis in 94/102 dogs (92.2%), nondiagnostic in 5/102 dogs (4.9%), not submitted in 1 dog, and not documented in 2 dogs. The diagnoses included acute or chronic hepatitis (33/102, 32.4%), vacuolar hepatopathy (26/102, 25.5%), neoplasia (21/102, 20.6%), cholangitis (5/102, 4.9%), and nodular hyperplasia (2/102, 1.9%) as described in Table 3. Seven of 102 (6.9%) dogs had other miscellaneous diagnoses (acute necrosis, eosinophilic vasculitis, vascular, normal).

**TABLE 3:** Summary of histopathologic diagnosis in dogs with and without major hemorrhage after PUGLB (n = 94)

Histopathologic diagnosis	Without major hemorrhage	With major hemorrhage
Chronic hepatitis (33/102)	(22/33) 66.7%	(11/33) 33.3%
Vacuolar hepatopathy (26/102)	(15/26) 57.7%	(11/26) 42.3%
Neoplasia (21/102)	(12/21) 57.2%	(9/21) 42.8%
Cholangitis (5/102)	(3/5) 60%	(2/5) 40%
Nodular hyperplasia (2/102)	(1/2) 50%	(1/2) 50%
Miscellaneous^a^ (7/102)	(7/7)100%	(0/7) 0%

Abbreviation: PUGLB, percutaneous ultrasound guided liver biopsy.

a Miscellaneous diagnoses included acute necrosis, eosinophilic vasculitis, vascular, and normal.

A total of 21/102 (20.6%) of dogs were diagnosed with neoplasia. The types of neoplasia diagnosed included hepatocellular carcinoma/adenoma (n = 11), sarcoma (n = 2), lymphoma (n = 2), metastatic carcinoma of unknown origin (n = 1), neuroendocrine tumor (n = 2), hepatobiliary adenocarcinoma (n = 1), metastatic islet cell tumor (n = 1), and round cell neoplasia of unknown type (n = 1).

There was no association between biopsy diagnosis and the risk of hemorrhage or occurrence of complications, except that dogs with neoplasia were more likely to have complications (*P* = .04). Both dogs with complications had neoplasia, 1 had lymphoma and the other multifocal hepatobiliary carcinoma.

## 5 DISCUSSION

This data suggest that in dogs, PUGLB is associated with a relatively high incidence of major hemorrhage (42.2%), but a low incidence of complications (1.9%). Complications occurred more commonly in dogs with neoplasia. The ΔPCV was significantly positively correlated with PCV before biopsy. Conventional indicators of hypocoagulability (platelet count, PT and aPTT), hepatobiliary disease severity (serum activity of liver‐derived enzymes, bilirubin and albumin concentrations), ultrasonographic appearance of the liver, sex, weight, and attributes around sample acquisition (needle size, number, experience of operator) did not predict the occurrence of complications or the magnitude of change in PCV after biopsy.

In humans a ΔPCV of 6% (ie, hemoglobin drops of >2 g/dL) occurs in about 7% of the population after a percutaneous liver biopsy.^4^, ^5^, ^6^, ^16^, ^17^, ^20^ In our study, 42.2% of the dogs undergoing PUGLB had a decrease in PCV greater than 6%. Thus, there seems to be a much higher incidence of asymptomatic hemorrhage in dogs after PUGLB compared to humans. The same has recently been shown in cats where 56.7% of cats had major hemorrhage after PUGLB.^27^ The reasons for these discrepancies between dogs and cats and humans are not immediately obvious. An additional 20 dogs became anemic after biopsy due to blood loss. Although seemingly asymptomatic, this blood loss can have clinical relevance, as it has been shown in critically ill dogs that hospital acquired anemia is associated with decreased survival to discharge.^28^

In a previous study of percutaneous ultrasound biopsy of abdominal and thoracic organs, 4.2% of dogs had a major complication (defined as requiring an intervention such as a transfusion) and 18.5% of dogs had a minor complication which was defined as a relative Hct decrease >10% with no intervention needed.^12^ Applying the same criteria as in this earlier study, the rate of both major complications (1.9%) and minor complications (15.4%) in the current study were slightly lower.^12^ In the former study, the inclusion of data from the biopsy of organs other than the liver, particularly the kidney, might explain the discrepancy in the results.^12^

Studies in dogs undergoing laparoscopic liver biopsy have shown complication rates of 4% and 1.8%, similar to that seen in the current study.^29^, ^30^ These laparoscopic studies however, did not report ΔPCV, so the incidence of asymptomatic major hemorrhage between the laparoscopic and percutaneous routes cannot be compared. Results however, suggest that complications with the acquisition of hepatic biopsies via percutaneous ultrasound guidance or by laparoscopy are rare events. The incidence of major hemorrhage after hepatic biopsy via laparoscopy, however, remains to be determined.

Although in this study PUGLB had a similar incidence of complications as laparoscopic liver biopsy, the practitioner needs to keep in mind that the accuracy of the former may be considerably less than the latter.^31^, ^32^ This is particularly true in congenital, inflammatory or copper associated diseases where the considerable variation in pathology between liver lobes necessitate obtaining multiple samples.^15^, ^31^, ^33^ Despite the lower accuracy of PUGLB, these tissue samples do provide valuable diagnostic information while being less invasive and less costly than surgical approaches.

In the current study, the only risk factor identified for hemorrhage after PUGLB was the presence of a higher PCV before biopsy. This is contrary to another study in which a large number of PUGLBs were evaluated and the mean PCV before biopsy was lower in dogs with major complications compared to dogs with no complications. ^12^ This is also contrary to a study on hemorrhage after laparoscopic biopsy, where 3 of the 4 dogs requiring a transfusion after the procedure were anemic before surgery.^30^ Anemia before biopsy is also a risk factor for complications in both cats and people undergoing PUGLB.^3^, ^27^

In humans, malignancy is a risk factor for major hemorrhage after PUGLB.^3^, ^4^ There were only 2 dogs with complications in our study and both of those dogs had neoplasia. Although we report that this was statistically significant based on a Fisher's exact test, this finding is limited by the power of our study. Considering the small number of dogs that had a complication, this could represent a Type 1 (alpha) error. Further studies, with a greater statistical power, to include a larger population of dogs diagnosed with hepatic neoplasia will be necessary to confirm that neoplasia is truly a risk factor.

Prothrombin time, aPTT and platelet count were not significantly different in dogs with and without major hemorrhage and there was no correlation between these coagulation variables and complications. This is in contrast to previous studies which show that dogs undergoing PUGLB and laparoscopic liver biopsy have an increased risk of complications with any prolongation in PT.^12^, ^29^ The reason for the difference could be due to the small number of dogs in the current study and the mild degree of PT prolongations observed. Also, PUGLB might not have been pursued in patients that had moderate to severely prolonged PT, aPTT, or both, given the potential risk of hemorrhage. This could have led to an overrepresentation of dogs with only mild coagulopathies observed in our study.

Alternatively, the poor correlation between PT and aPTT with actual clinical hemorrhage could be due to the failure of these tests to measure the complex coagulation abnormalities that can exist in liver disease. In humans with liver disease there is some evidence that serum fibrinogen and whole blood techniques such as thromboelastography may be better at predicting hemorrhage from provocative procedures.^23^, ^34^, ^35^, ^36^ Studies looking at thromboelastography in dogs with chronic hepatitis and acute liver failure have defined populations of dogs that are hypocoagulable, hyperfibrinolytic, or both, that might be at risk of hemorrhage.^37^, ^38^ Prospective studies looking at a more extensive array of coagulation parameters such as serum fibrinogen and thromboelastography in dogs undergoing PUGLB are necessary.

Limitations of this study include the retrospective nature and small sample size. In addition, it was difficult to discern the total fluids administered intravenously between the time of biopsy and PCV assessment after the biopsy from the medical records. If substantial, this fluid might have promoted hemodilution and a lower PCV, although it is not our hospital's practice to pursue a sedated biopsy procedure on hemodynamically unstable dogs. Most dogs were under anesthesia for the biopsy procedure which can lead to vasodilation and fluid shifts that can lower the PCV. Typically these effects are gone by 2 hours after anesthesia and the average time to the lowest PCV in this study was 4 hours after biopsy.^39^ Based on the information available in the records, the need for resuscitative fluid, transfusion, or both, was most likely related to hemorrhage, although because of the retrospective nature of the study, dogs were not followed closely to identify other causes of volume depletion or hypovolemia. There was limited assessment of coagulation (only platelet count, PT, and aPTT were inclusion criteria) in the current study. In addition, very few dogs had serious alterations in coagulation status, which could have affected the results of the study. The low complication rate made it hard to find significant associations. Concurrent tissue sampling of other organs could have influenced the incidence of hemorrhage. It was difficult to differentiate, especially in this subset of dogs, the extent of their contribution to hemorrhage, leading to reduction in PCV after biopsy.

## 6 CONCLUSIONS

Percutaneous ultrasound guided liver biopsy in the population of dogs in the current study, with normal or mild coagulation abnormalities, results in a high incidence of clinically silent, major hemorrhage (42.5%), but complications (1.9%) are rare. There is a poor correlation between conventional coagulation tests and severity of hemorrhage or the occurrence of complications. Investigation into other methods to predict hemorrhage after PUGLB are needed. The data from the current study should help inform power calculations for future studies examining risk factors for complications associated with PUGLB in dogs.

## CONFLICT OF INTEREST DECLARATION

Authors declare no conflict of interest.

## OFF‐LABEL ANTIMICROBIAL DECLARATION

Authors declare no off‐label use of antimicrobials.

## INSTITUTIONAL ANIMAL CARE AND USE COMMITTEE (IACUC) OR OTHER APPROVAL DECLARATION

Authors declare no IACUC or other approval was needed.

## HUMAN ETHICS APPROVAL DECLARATION

Authors declare human ethics approval was not needed for this study. Medical records from the Foster Hospital for Small Animals at Cummings School of Veterinary Medicine at Tufts University between 2002 and 2015.
